# Increasing survival gap between young and elderly gastric cancer patients

**DOI:** 10.1007/s10120-017-0708-7

**Published:** 2017-03-09

**Authors:** S. D. Nelen, R. H. A. Verhoeven, V. E. P. P. Lemmens, J. H. W. de Wilt, K. Bosscha

**Affiliations:** 1 Department of Surgery, Radboud University Medical Center, Geert Grooteplein 10, Route 618, P.O. 9101, 6500 HB Nijmegen, The Netherlands; 2 Department of Research, Netherlands Comprehensive Cancer Organization (IKNL), Utrecht, The Netherlands; 3 Department of Public Health, Erasmus University Medical Centre, Rotterdam, The Netherlands; 4 Department of Surgery, Jeroen Bosch Hospital, ‘s-Hertogenbosch, The Netherlands

**Keywords:** Stomach neoplasms, Surgery, Chemotherapy, Epidemiology, Curative

## Abstract

**Introduction:**

This study investigates the treatment and survival of young versus elderly potentially curable gastric cancer patients in the Netherlands.

**Patients and methods:**

All noncardia gastric cancer patients with potentially curable gastric cancer according to stage (cTx–3, cNx–3, and cMx–0) diagnosed between 1989 and 2013 were selected from the Netherlands Cancer Registry. Trends in treatment and overall survival were compared between young patients (younger than 70 years) and elderly patients (70 years or older). Multivariable logistic regression analysis was used to examine the probability of patients undergoing surgery and chemotherapy in the most recent period. Multivariable Cox regression analysis was used to identify independent factors associated with survival.

**Results:**

In total, 8107 young and 13,814 elderly gastric cancer patients were included. There was a major increase in the proportion of patients treated with resection and chemotherapy after 2004–2008. In young patients the increase was from 2.6% in 1999–2003 to 63% in 2009–2013 (*p* < 0.01). Also an increase was noticed among elderly patients, from 0.1% to 16% (*p* < 0.01). Median survival increased from 2004 to 2008 onward particularly in young patients and to a lesser extent in elderly patients (from 28 to 41 months vs from 11 to 13 months). Multivariable Cox regression analyses confirmed that overall survival improved for young and elderly patients.

**Discussion:**

Young patients experienced a stronger improvement in survival than elderly patients, resulting in an increasing survival gap. The literature shows this is a problem not only in the Netherlands but also throughout Europe. The dissimilarity in treatment between young and elderly patients could be the reason for this difference.

## Introduction

Gastric cancer is a disease of the elderly, with almost 60% of all patients being older than 70 years in 2015 [1]. Since elderly patients are often not included in clinical trials, evidence-based guidelines are mainly based on the results of treatment in young patients. Therefore, the best treatment in the fit or frail elderly with gastric cancer and the distinction with young patients remain unclear [2–4].

The care for elderly gastric cancer patients compared with young patients differs in various aspects. One of the most important differences is the large and growing prevalence of comorbidity in elderly people; 72% of male patients older than 80 years have comorbidity [5]. As a result, elderly patients are considered less often to be able to undergo a (partial) gastrectomy, even though a curative resection offers the only chance for cure. The high prevalence of comorbidity also results in increasing 30-day postoperative mortality rates, being more than 10% in elderly gastric cancer patients (74 years or older) compared with less than 5% for patients younger than 65 years [5, 6]. Furthermore, studies have suggested that elderly gastric cancer patients often have a more advanced stage of disease and a larger proportion of cardia cancers, which are associated with a poorer prognosis [7–9]. Among other reasons, this might have led to the increasing survival difference between older and young patients in Europe in a previous study [10].

The outcome for gastric cancer patients remains poor, and until recently the 5-year relative survival rate of gastric cancer patients had not increased in any age group [11–14]. To improve survival, the treatment of gastric cancer patients in the Netherlands has changed in recent years. This included centralization of gastric cancer surgery with a minimal annual volume of 20 gastrectomies, and an increased use of (neo)adjuvant chemotherapy.

It is unclear if survival has improved through changing management of gastric cancer in the Netherlands, and it is not known to what extent the variation in treatment between young and elderly patients resulted in an age-related survival difference. This study, therefore, investigates treatment patterns and survival for young and elderly patients with potentially curable gastric cancer in the Netherlands.

## Methods

Data were obtained from the population-based Netherlands Cancer Registry (NCR). This registry serves the total Dutch population of approximately 17 million inhabitants. The NCR is based on notification of all newly diagnosed malignancies in the Netherlands by the national automated pathological archive (PALGA). Additional sources are the national registry of hospital discharge and radiotherapy institutions. Information on diagnosis, staging, and primary treatment is routinely extracted from the medical records by specially trained data managers of the NCR. The information on vital status is obtained by an annual linkage with the municipal administrative databases, which register all deceased persons in the Netherlands and all persons who have emigrated from the Netherlands.

Topography and morphology were coded according to the International Classification of Diseases for Oncology, third edition [15]. The distribution of the location in the stomach was divided as follows: proximal/middle [fundus, corpus, lesser and greater curvature (C16.1, C16.2, C16.5, and C16.6)], pyloric and antrum (C16.3, C16.4), and overlapping or not otherwise specified (C16.8, C16.9). Tumor staging was performed according to different versions of Union for International Cancer Control (UICC) TNM classification, but all records were recoded to the fifth UICC TNM classification [16]. For clinical UICC TNM N classification, we used only N0 or N+, since the reliability of evaluating the exact preoperative N category is low [17]. To calculate tumor stage, the pathological TNM classification was used; if this was unknown, a clinical TNM classification was used.

For this study we selected all patients with noncardia gastric cancer diagnosed in the period 1989–2013 who had no clinically distant metastases (UICC TNM classification cM0 or cMx). Tumor size was limited to tumors penetrating up to the serosa without invasion of adjacent structures on clinical examination (cT0-3 or cTx according to the fifth UICC TNM classification). These patients were considered as potentially curable according to their clinical TNM classification. Patients were analyzed per age group (younger than 70 years vs 70 years or older) according to the limits proposed by the European Society of Medical Oncology and the Dutch clinical guidelines [5, 18].

### Statistical analysis

Descriptive statistics were used to characterize the patients the young and elderly patients. Bar graphs were drawn to assess the variation in treatment modalities and tumor stage throughout the years for young and elderly patients. Univariable and multivariable logistic regression analyses were performed for young and elderly patients to examine the influence of different clinicopathological factors with regard to patients undergoing surgery and chemotherapy in the period from 2009–2013. As the treatment paradigm for gastric cancer patients changed over time, we decided that investigating the factors related to treatment decisions was most interesting for recent patients. Therefore, the logistic regression analyses were limited to patients in whom gastric cancer had been diagnosed in the period 2009–2013.

Survival time was defined as the time from diagnosis to death or until February 1, 2016 for patients who were still alive. Kaplan–Meier curves were generated to examine the overall survival for young and elderly patients over sequential periods. The difference in median overall survival between young and elderly patients over time was calculated. Multivariable Cox regression analyses were performed for young and elderly patients to investigate the influence of various patient-, tumor-, and treatment-specific variables on overall survival over time. The results from survival analyses using Cox regression analyses were reported as hazard ratios and the 95% confidence interval. Reported *p* values less than 0.050 were considered statistically significant. All analyses were conducted with IBM SPSS Statistics version 23.

## Results

The number of patients with potentially curable noncardia gastric cancer compared with all patients with noncardia gastric cancer is given in Table 1. In total, 21,867 patients (58%) with potentially curable noncardia gastric cancer were selected from 38,004 patients with noncardia gastric cancer. Not only the total number of patients decreased but also the percentage of young patients with potentially curable noncardia gastric cancer decreased from 57% in 1989–1993 to 48% in 2009–2013, whereas in the elderly patients the decreased was from 63% to 57%.

**Table 1:** The number of all noncardia gastric cancer patients and the number of patients with potentially curable gastric cancer (cTx–3, cNx–3, cMx–0) in the Netherlands according to age group and period of diagnosis

	Young (<70 years)		Elderly (≥70 years)		Total	
	All	Curable	All	Curable	All	Curable
1989–1993	3825	2191 (57%)	5482	3467 (63%)	9307	5658 (60%)
1994–1998	3209	1758 (55%)	4881	3013 (62%)	8090	4771 (59%)
1999–2003	2916	1481 (51%)	4425	2710 (61%)	7341	4191 (57%)
2004–2008	2657	1354 (51%)	4038	2383 (59%)	6695	3737 (55%)
2009–2013	2676	1294 (48%)	3895	2216 (57%)	6571	3510 (54%)
Total	15,283	8095 (53%)	22,721	13,789 (61%)	38,004	21,867 (58%)

The patient and tumor characteristics of the patients with potentially curable disease are presented in Table 2. Compared with younger patients, elderly patients were more often female. The tumor location of elderly patients was more often specified as not otherwise specified or overlapping (27% vs 32%, *p* < 0.05). Young patients more often had a diagnosis of a poorly or undifferentiated tumor (55% vs 47%, *p* < 0.05) or a signet cell carcinoma (25% vs 15%, *p* < 0.05). Elderly patients more often underwent no resection or no chemotherapy (39%) in comparison with the young patients (10%, *p* < 0.05) and as a result their cancer was more often staged as an unknown pathological T, N, and M category.

**Table 2:** Patient characteristics of patients with potential curable (cTx–3, cNx–3, cMx–0) noncardia gastric cancer diagnosed between 1989 and 2013 by age

		Young (<70 years), *N* = 8095		Elderly (≥70 years), *N* = 13,789		Total, *N* = 21,867	
		*n*	%	*n*	%	*n*	%
Sex	Male	5152	64	7733	56	12,885	59
	Female	2926	36	6056	44	8982	41
pT category	0/1	1404	17	1509	11	2913	13
	2	2940	36	3796	28	6736	31
	3	2062	26	2393	17	4455	20
	4	395	4.9	415	3.0	810	3.7
	Unknown	1227	16	5676	41	6903	32
pN category	0	2592	32	3025	22	5617	26
	1	2405	30	2793	20	5198	24
	2	1023	13	1050	7.6	2073	9.5
	3	247	3.1	250	1.8	497	2.3
	Unknown	1811	22	6671	48	8482	39
pM category	0	3819	47	4651	34	8470	39
	1	280	3.5	271	2.0	551	2.5
	Unknown	3797	49	8867	64	12,664	58
Tumor location	Proximal and middle	2621	32	3866	28	6487	30
	Fundus and antrum	3260	40	5465	40	8725	40
	NOS and overlapping	2214	27	4471	32	6685	31
Tumor grade	Well differentiated	266	3.3	517	3.7	783	3.6
	Moderately differentiated	1444	18	3263	24	4707	22
	Poorly differentiated and undifferentiated	4466	55	6545	47	11,011	50
	Unknown	1919	24	3477	25	5396	25
Tumor morphology	Adenocarcinoma	5162	64	10,614	77	15,776	72
	Nonadenocarcinoma	389	4.8	558	4.0	947	4.3
	Linitis plastica	504	6.2	579	4.2	1083	5.0
	Signet ring cell carcinoma	2040	25	2051	15	4091	19
Treatment group	No resection, no chemotherapy	813	10	5425	39	6238	29
	Resection	5698	70	7733	56	13431	61
	Chemotherapy	320	4.0	174	1.3	494	2.3
	Resection and chemotherapy	1264	16	470	3.4	1734	7.9
Hospital of diagnosis	University	638	7.9	751	5.4	1389	6.4
	Training hospital	4771	59	8186	59	12,957	59
	Nontraining hospital	2669	33	4852	35	7521	34

The mean age of the young patients was 59.0 years (range 13–69 years), that of the elderly patients was 79.4 years (range 70–103 years), and that of all patients was 71.9 years (13–103 years). For all variables, *p* < 0.05

*NOS* not otherwise specified

**Fig. 1:**
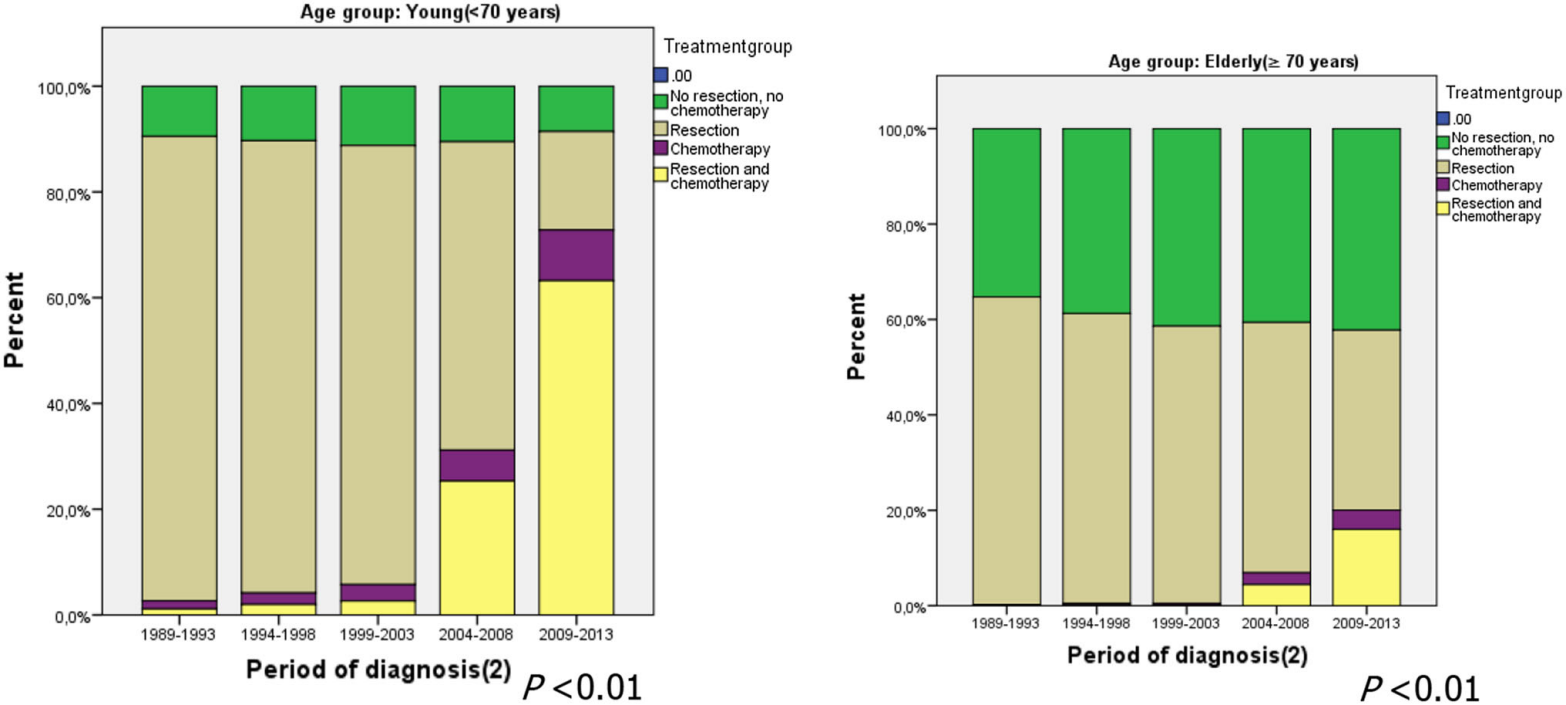
Treatment modality according to period of diagnosis for young patients (*left*) and elderly patients (*right*) with potentially curable noncardia gastric cancer

The proportion of young patients undergoing neither surgery nor chemotherapy remained stable over sequential years (9.5% vs 8.6%, *p* = 0.23). For elderly patients the percentage increased significantly: between 1989 and 1993, 35% of all elderly patients did not undergo surgery or chemotherapy, but the proportion increased to 42% in the period 2009–2013 (*p* < 0.01) (Fig. 1). In both the young and the elderly patients there was a strong increase in the proportion of patients treated with resection and chemotherapy after 2004–2008; in young patients the proportion increased from 2.6% in 1999–2003 to 63% in 2009–2013 (*p* < 0.01), and in elderly patients the increase was from 0.1% to 16% (*p* < 0.01).

**Fig. 2:**
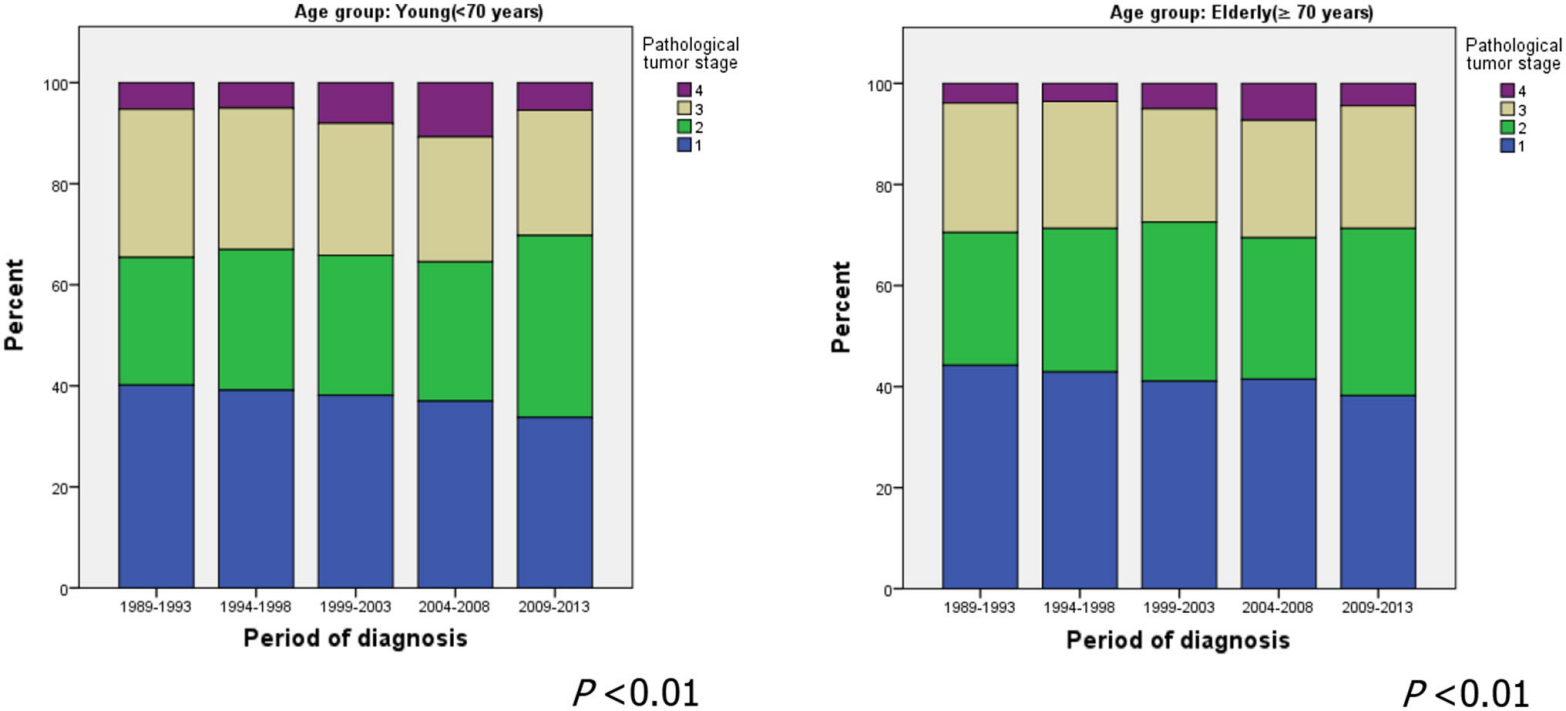
Pathological tumor stage distribution by period of diagnosis for young patients (*left*) and elderly patients (*right*) with potentially curable noncardia gastric cancer

There were only minor differences in tumor stage distribution through the years. A shift from stage I to stage II was seen in both young and elderly patients with gastric cancer. Between 1989 and 1993, 40% of all young gastric cancer patients had stage I disease and 25% had stage II disease. Between 2009 and 2013, the proportion with stage I disease decreased to 34% and the proportion with stage II disease increased toward 36%. A similar trend was seen in elderly patients (Fig. 2).

The results of the multivariable logistic regression analyses on the chance of being treated with resection and chemotherapy in the period 2009–2013 are presented in Table 3. The factors that significantly influenced the chances of patients undergoing chemotherapy and resection were mostly the same for the young and the elderly patients. In both age groups there was a decreased chance of patients undergoing chemotherapy and surgery with increasing age and lower cT category (cT0/cT1 vs cT2). Also in young patients, patients with tumors with a favorable histological grade, overlapping or not otherwise specified tumors, and a diagnosis in an academic hospital had a lower chance of undergoing chemotherapy and surgery. Elderly patients with a tumor of the fundus or antrum had a significant lower chance of receiving both treatment options.

**Table 3:** Multivariable logistic regression analysis on the influence of different clinicopathological factors on the probability of undergoing surgery and chemotherapy between 2009 and 2013 for young and elderly patients with potentially curable noncardia gastric cancer

		Young (<70 years)			Elderly (≥70 years)		
		OR	95% CI	*p*	OR	95% CI	*p*
Sex	Male	Reference			Reference		
	Female	0.93	0.73–1.20	0.58	0.84	0.62–1.12	0.23
Age		0.97	0.96–0.99	*<0.01*	0.74	0.71–0.76	*<0.01*
Year of diagnosis	2009	Reference			Reference		
	2010	0.81	0.55–1.20	0.30	1.17	0.74–1.86	0.50
	2011	1.09	0.73–1.62	0.68	1.29	0.82–2.03	0.27
	2012	1.01	0.69–1.49	0.94	1.22	0.77–1.94	0.40
	2013	0.98	0.66–1.46	0.94	1.33	0.84–2.11	0.22
cT category	0/1	0.18	0.10–0.32	*<0.01*	0.32	0.16–0.65	*<0.01*
	2	Reference			Reference		
	3	0.67	0.36–1.26	0.22	1.10	0.50–2.42	0.81
	Unknown	0.64	0.49–0.84	*<0.01*	0.60	0.45–0.81	*<0.01*
cN category	0	Reference			Reference		
	cN1–3	1.08	0.82–1.44	0.58	1.14	0.83–1.58	0.41
	Unknown	0.54	0.38–0.77	*<0.01*	0.43	0.27–0.68	*<0.01*
Tumor morphology	Adenocarcinoma	Reference			Reference		
	Nonadenocarcinoma	1.13	0.73–1.73	0.58	0.87	0.51–1.49	0.61
	Linitis plastica	0.92	0.56–1.53	0.76	0.71	0.37–1.35	0.30
	Signet ring cell carcinoma	1.06	0.78–1.44	0.69	1.16	0.81–1.65	0.42
Tumor grade	Well differentiated	0.30	0.11–0.82	*0.02*	0.46	0.15–1.45	0.19
	Moderately differentiated	0.56	0.38–0.84	*0.01*	0.66	0.44–1.01	0.06
	Poorly differentiated and undifferentiated	Reference			Reference		
	Unknown	1.01	0.78–1.31	0.96	1.15	0.85–1.56	0.36
Tumor location	Proximal and middle	Reference			Reference		
	Fundus and antrum	0.89	0.67–1.18	0.42	0.57	0.41–0.79	*<0.01*
	NOS and overlapping	0.60	0.43–0.85	*<0.01*	0.79	0.55–1.12	0.18
Diagnosing hospital	University	0.40	0.26–0.62	*<0.01*	0.60	0.33–1.07	0.08
	Training hospital	Reference			Reference		
	Nontraining hospital	1.16	0.89–1.51	0.27	1.12	0.83–1.50	0.46

Values in *italic* are statistically significant

*CI* confidence interval, *NOS* not otherwise specified, *OR* odds ratio

**Fig. 3:**
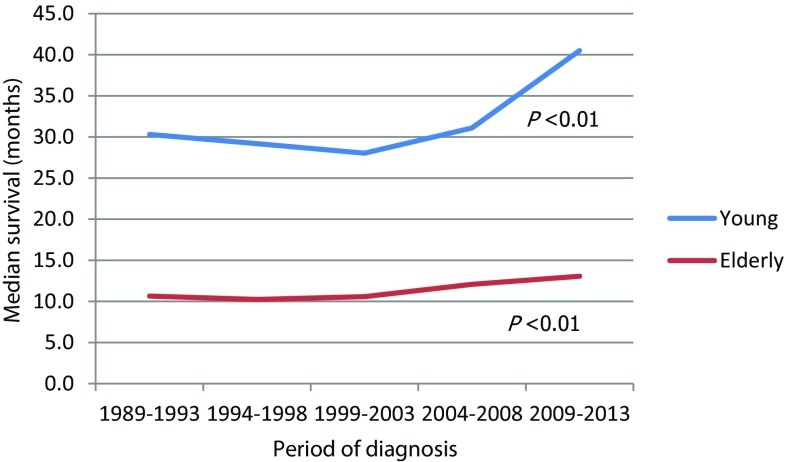
Median survival per period of diagnosis for patients with potentially curable noncardia gastric cancer and the difference between both age groups

The median overall survival over time for both age groups of patients treated with curative intent is presented in Fig. 3. The median survival of patients younger than 70 years increased from 30.3 months between 1989 and 1993 to 40.5 months between 2009 and 2013 (*p* < 0.01). For elderly patients, the median survival increased from 10.7 months to 13.1 months (*p* < 0.01). Additionally performed survival analyses on all noncardia gastric cancer patients (i.e., patients treated with curative intent and palliative intent) in the Netherlands between 1989 and 2013 showed a significant improvement of overall survival for all gastric cancer patients from 7.6 months between 1989 and 1993 to 8.4 months between 2009 and 2013 (*p* < 0.01) (data not shown).

**Fig. 4:**
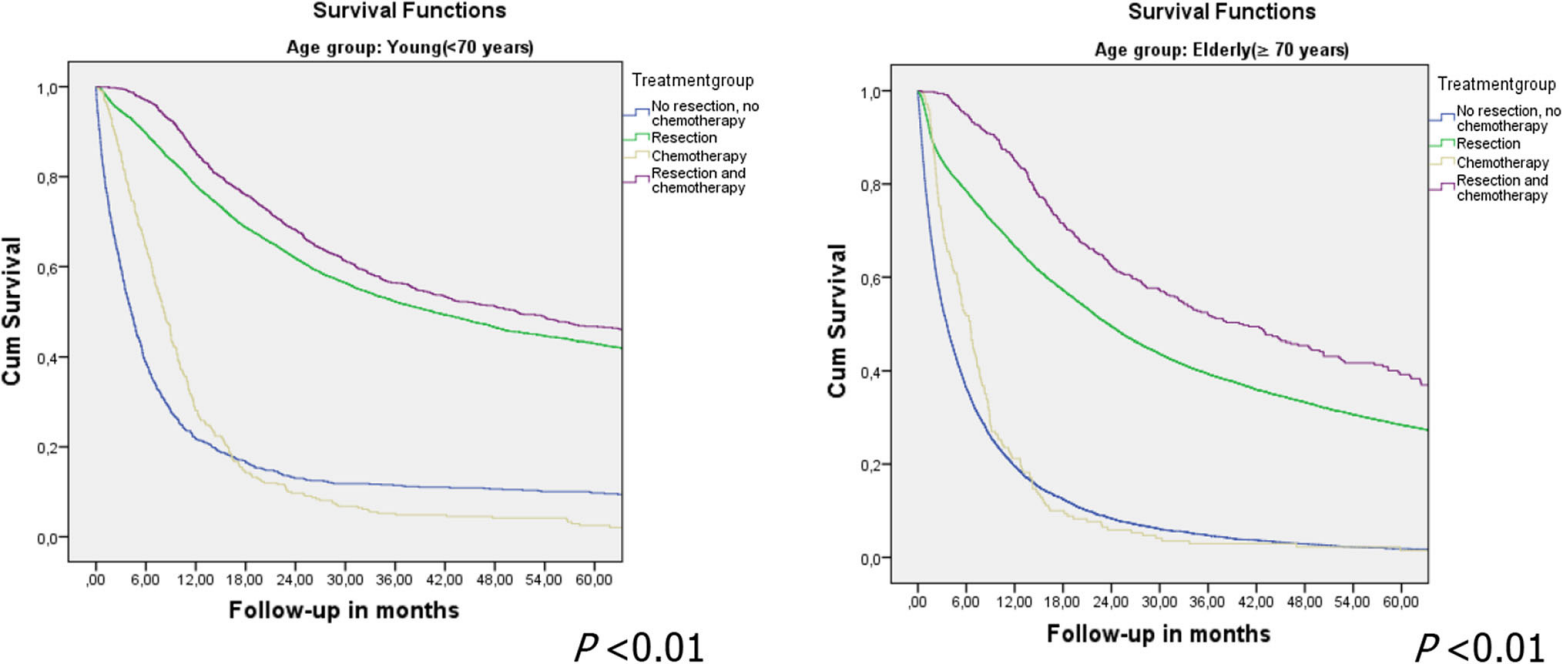
Kaplan–Meier 5-year overall survival curve per treatment method for young patients (*left*) and elderly patients (*right*) with potentially curable noncardia gastric cancer

Patients with potentially curable gastric cancer who underwent surgery and chemotherapy had the best 5-year overall survival rates of 47% and 39% for the young and elderly patients respectively (Fig. 4). Patients who did not undergo resection or chemotherapy or who underwent only chemotherapy had the worst survival.

Multivariable Cox regression confirmed the improved survival over time (Table 4). In young patients survival improved after 2004–2008, with a hazard ratio of 0.74 (95% confidence interval 0.65–0.83) in 2009–2013 compared with 1989–1993. For the elderly patients survival improved after 1999–2003, with a hazard ratio of 0.85 (95% confidence interval 0.80–0.90). Other factors that significantly affected overall survival were sex, age, pathological T, N, and M category, tumor location, morphology, and grade, treatment, and type of diagnosing hospital, with only limited differences between young and elderly patients.

**Table 4:** Cox regression analyses on the influence of different clinicopathological factors on overall survival in young and elderly patients with potentially curable noncardia gastric cancer

		Young (<70 years)			Elderly (≥70 years)		
		HR	95% CI	*p*	HR	95% CI	*p*
Sex	Male	Reference			Reference		
	Female	0.92	0.88–0.98	*<0.01*	0.83	0.80–0.86	*<0.01*
Age		1.03	1.02–1.03	*<0.01*	1.03	1.02–1.03	*<0.01*
Period of diagnosis	1989–1993	Reference			Reference		
	1994–1998	1.01	0.94–1.08	0.82	1.01	0.96–1.06	0.69
	1999–2003	0.97	0.90–1.05	0.46	0.90	0.85–0.95	*<0.01*
	2004–2008	0.90	0.83–0.99	*0.02*	0.85	0.80–0.90	*<0.01*
	2009–2013	0.74	0.65–0.83	*<0.01*	0.72	0.67–0.76	*<0.01*
pT category	0/1	0.36	0.33–0.40	*<0.01*	0.56	0.51–0.60	*<0.01*
	2	Reference			Reference		
	3	0.72	0.67–0.77	*<0.01*	0.78	0.73–0.82	*<0.01*
	4	1.32	1.17–1.49	*<0.01*	1.35	1.20–1.51	*<0.01*
	Unknown	1.14	0.97–1.33	0.11	1.32	1.17–1.50	*<0.01*
pN category	0	Reference			Reference		
	1	1.74	1.62–1.86	*<0.01*	1.54	1.46–1.64	*<0.01*
	2	2.28	2.09–2.49	*<0.01*	2.09	1.94–2.26	*<0.01*
	3	2.98	2.54–3.49	*<0.01*	2.42	2.09–2.80	*<0.01*
	Unknown	1.08	0.96–1.21	0.21	1.18	1.09–1.27	*<0.01*
pM category	0	Reference			Reference		
	1	1.92	1.69–2.18	*<0.01*	1.83	1.61–2.07	*<0.01*
	Unknown	0.96	0.90–1.02	0.16	1.05	1.00–1.10	*0.05*
Tumor location	Proximal and middle	Reference			Reference		
	Fundus and antrum	1.09	1.03–1.16	*0.01*	1.04	1.00–1.09	0.05
	NOS and overlapping	1.26	1.18–1.35	*<0.01*	1.23	1.17–1.28	*<0.01*
Tumor morphology	Adenocarcinoma	Reference			Reference		
	Nonadenocarcinoma	1.25	1.10–1.41	*<0.01*	1.20	1.09–1.31	*<0.01*
	Linitis plastica	1.79	1.61–1.99	*<0.01*	1.47	1.34–1.60	*<0.01*
	Signet ring cell carcinoma	1.07	1.00–1.14	*0.05*	1.11	1.05–1-16	*<0.01*
Tumor grade	Well differentiated	1.03	0.90–1.20	0.65	0.84	0.76–0.92	*<0.01*
	Moderately differentiated	0.90	0.84–0.97	*<0.01*	0.90	0.86–0.94	*<0.01*
	Poorly differentiated and undifferentiated	Reference			Reference		
	Unknown	0.94	0.88–1.01	0.08	0.86	0.82–0.90	*<0.01*
Treatment	No resection, no chemotherapy	Reference			Reference		
	Resection	0.25	0.21–0.30	*<0.01*	0.42	0.37–0.48	*<0.01*
	Chemotherapy	0.92	0.80–1.05	0.21	0.99	0.85–1.16	0.93
	Resection and chemotherapy	0.20	0.17–0.25	*<0.01*	0.30	0.25–0.36	*<0.01*
Diagnosing hospital	University	0.79	0.71–0.88	*<0.01*	0.84	0.77–0.91	*<0.01*
	Training hospital	Reference			Reference		
	Nontraining hospital	0.97	0.92–1.02	0.26	1.03	1.00–1.07	0.08

Values in *italic* are statistically significant

*CI* confidence interval, *HR* hazard ratio, *NOS* not otherwise specified

## Discussion

This study is the first study to show a significantly improved overall survival for patients with potentially curable noncardia gastric cancer in the Netherlands in recent years. Improvement of overall survival was more pronounced in young patients, which led to an increasing survival difference between young and elderly patients with potentially curable noncardia gastric cancer. Both young and elderly patients were increasingly treated with surgery and chemotherapy.

A lack of improvement in survival was reported in various previous studies throughout the world. Previous Dutch and Japanese studies did not show any improvement of survival for stage I–III gastric cancer patients [11, 13, 14, 19]. In the present study, median overall survival improved in potentially curable patients by 34% in young patients and 23% in elderly patients between 1989 and 2013, which was comparable with the findings of a large European study [20].

There could be several possible explanations for the improvement of survival in potentially curable noncardia gastric cancer patients. First, it might be caused by stage migration, because patients who previously received a diagnosis of nonmetastasized disease might nowadays receive a diagnosis of metastasized disease possibly due to the improvement of preoperative diagnostic imaging. This would consequently lead to a group of potentially curable patients with a better prognosis. Nevertheless, this study showed that survival of all noncardia gastric cancer patients (both nonmetastasized and metastasized) also improved significantly, which indicates that stage migration cannot be the only cause for the improvement in survival.

Another additional explanation for the improvement of overall survival in potentially curable noncardia gastric cancer patients could be the effect of centralization of gastric cancer surgery in the Netherlands. Although the beneficial effect of centralization of gastric cancer surgery on long-term survival has not been proven so far, it is thought to improve outcome after gastrectomy [21, 22]. From 2012 gastrectomies in the Netherlands were centralized to a minimum of ten per hospital per year, and as of 2013 this has been increased to a minimum of 20 per year. In this study, overall survival already started to improve before 2012, indicating that the introduction of the minimal annual volume norm is also not the only cause for improvement of survival but it could partly explain the survival improvement in recent years.

The Magic trial reported better survival rates for patients who underwent perioperative chemotherapy and surgery compared with surgery alone for gastric cancer. Since 2006 chemotherapy has been increasingly used in the Netherlands [13, 23]. Since then two meta-analyses on the effect of adjuvant and neoadjuvant chemotherapy on overall and disease-free survival have demonstrated a positive effect on survival [24, 25]. In the present study a strong increase in the use of chemotherapy was observed in the Netherlands, in particular in young patients. This increased use of perioperative chemotherapy might be an important factor in the improved survival demonstrated in recent years.

Apart from increased overall survival in young and elderly patients, the present study showed an increasing survival gap between young and elderly patients. When these results are put into a European perspective, a similar result was observed in a large number of population-based cancer registries throughout Europe [10, 26, 27]. The EUROCARE IV and EUROCARE V studies as well as the EUROCARE study on age-related survival differences showed not only a significantly improved overall survival but also an increased survival difference between young and elderly patients [10, 26, 27].

The present study, which was done later than the EUROCARE studies, observed an even greater increase in the overall survival difference between young and elderly patients compared with the EUROCARE studies, especially in recent years. Among other reasons, this might be caused by differences in treatment between young and elderly patients. Noticeable is the proportion of patients who did not undergo surgery or chemotherapy. In young patients, the proportion of patients has remained stable at around 10% in the last 20 years. For elderly patients, the proportion has increased significantly from 35% to 42% in the same period. An explanation for this increasing percentage might be the better understanding of age-related differences in treating gastric cancer. Many studies have reported potential risks of surgery and/or chemotherapy in elderly patients, which could have stopped clinicians from treating patients older than 70 years [28, 29].

On the other hand, the percentage of patients undergoing both surgery and chemotherapy increased in both age groups, but to a much lesser extent in elderly patients. Patients treated with both chemotherapy and resection have the highest survival rate in the present study. However, there most likely is a selection bias, as fitter patients are likelier to be treated more extensively.

It remains unclear whether omitting any form of treatment is caused by good clinical judgment or is because of the clinicians’ assumption that chronological age makes elderly patients unfit for therapy [10]. Previous studies based on data from the NCR showed a large variation in the probability of patients undergoing surgery, ranging from 53% to 83% according to hospital diagnoses between 2005 and 2013 [12, 30]. This suggests that the treatment regimen is determined not only by patient- and/or disease-specific factors. This variation significantly impacted survival [30]. Furthermore, the chance of receiving (neo)adjuvant treatment also varied between regions. Depending on the region of treatment, the odds ratio of receiving (neo)adjuvant treatment ranged from 0.7 to 4.5 [12].

The limitations of this study are the lack of data on comorbidity, performance status, and the possible contributing reasons to forgo surgery or chemotherapy. These factors are known to impact treatment choice and survival. We were, however, unable to adjust the data for these factors in our analyses since these factors are not routinely collected by the NCR on a national level. The main strength of the study is the size of the study population and the fact that the study is based on nationwide population-based data, which makes it possible to investigate trends in treatment, survival, and the influence of various clinicopathological factors on treatment and survival, representing daily clinical practice.

In conclusion, overall survival of potentially curable noncardia gastric cancer patients has significantly improved in recent years. Especially young patients showed a strong improvement of survival, a trend that is seen throughout Europe [26, 27]. This strong improvement of overall survival could be caused by a higher proportion of patients undergoing both surgery and chemotherapy. Besides, further research will be needed to improve survival in (frail) elderly gastric cancer patients to prevent a further increase in the survival gap between young and elderly gastric cancer patients.
